# Prospective validation in epithelial tumors of a gene expression predictor of liver metastasis derived from uveal melanoma

**DOI:** 10.1038/s41598-019-52841-y

**Published:** 2019-11-20

**Authors:** Petros Tsantoulis, Mauro Delorenzi, Ivan Bièche, Sophie Vacher, Pascale Mariani, Nathalie Cassoux, Alexandre Houy, Marc-Henri Stern, Sergio Roman-Roman, Pierre-Yves Dietrich, Arnaud Roth, Wulfran Cacheux

**Affiliations:** 1 Hôpitaux Universitaires de Genève, Service d’Oncologie, Geneva, Switzerland; 2 University of Geneva, Geneva, Switzerland; 3 SIB Swiss Institute of Bioinformatics, Bioinformatics Core Facility, Lausanne, Switzerland; 4 University Lausanne, Department of Fundamental Oncology, Lausanne, Switzerland; 5 Ludwig Institute for Cancer Research, Epalinges, Switzerland; 6 Institut Curie, Département de génétique, Paris, France; 7 Institut Curie, Département de chirurgie, Paris, France; 8 Institut Curie, PSL Research University, INSERM U830 Paris, France; 9 Institut Curie, PSL Research University, Translational Research Department, Paris, France; 10 Hôpital Privé – Pays de Savoie, Oncology department, Annemasse, France

**Keywords:** Metastasis, Tumour biomarkers, Cancer genomics, Tumour biomarkers

## Abstract

Predicting the risk of liver metastasis can have important prognostic and therapeutic implications, given the availability of liver-directed therapy. Uveal melanoma has a striking predisposition for liver metastasis despite the absence of anatomical proximity. Understanding its biology may uncover factors promoting liver metastasis in other malignancies. We quantified gene expression by RNAseq in 76 uveal melanomas and combined with public data in a meta-analysis of 196 patients. The meta-analysis of uveal melanoma gene expression identified 63 genes which remained prognostic after adjustment for chromosome 3 status. Two genes, *PTP4A3* and *JPH1*, were selected by L1-penalized regression and combined in a prognostic score. The score predicted liver-specific relapse in a public pan-cancer dataset and in two public colorectal cancer datasets. The score varied between colorectal consensus molecular subtypes (CMS), as did the risk of liver relapse, which was lowest in CMS1. Additional prospective validation was done by real-time PCR in 463 breast cancer patients. The score was significantly correlated with liver relapse in hormone receptor positive tumors. In conclusion, the expression of *PTP4A3* and *JPH1* correlates with risk of liver metastasis in colorectal cancer and breast cancer. The underlying biological mechanism is an interesting area for further research.

## Introduction

Almost all patients with metastatic uveal melanoma present liver metastases^1^, making this rare disease an ideal model for the identification of biological features predisposing to liver metastasis. Our objective was to use the gene expression profile of uveal melanoma to identify genes that would predict the risk of liver metastasis in epithelial malignancies, especially colorectal cancer. Surgical resection of liver metastases from colorectal cancer can be performed with curative intent and is recognized as standard of care under certain conditions^2,3^. The optimal management of oligometastatic liver spread from other tumor types is not as clearly defined but curative treatment can be contemplated on a case-by-case basis. In addition to surgery, less invasive modalities such as radiofrequency ablation^4^, hepatic arterial infusion chemotherapy^5^ or local immunotherapy (trials NCT02509507 and NCT03256344) are being explored for patients with metastases limited to the liver. In conclusion, the identification of patients at high risk of liver metastasis could justify more aggressive treatment up-front (for example, adjuvant chemotherapy) and more intensive liver-specific follow-up with the intention to detect and treat liver metastases as early as possible with an appropriate treatment modality.

Several prognostic features have been identified in uveal melanoma and are associated with the development of distal metastasis, typically in the liver. Monosomy of chromosome 3 often co-occurs with mutation of the *BAP1* gene in 3p21.1, results in bi-allelic inactivation and is a hallmark of uveal melanoma with unfavorable prognosis^6^. However, the *BAP1* mutation is rare in other cancer types (less than 5%), with the exception of kidney cancer^7^, mesothelioma^8^ and cholangiocarcinoma^9^. Gain of chromosome arm 8q is also associated with unfavorable prognosis in uveal melanoma^10–12^, as is overexpression of *PTP4A3*, which resides in this arm, specifically in 8q24.3^13^. In contrast with the *BAP1* mutation, gain of 8q is a frequent event in many cancer types, including colorectal^14^, gastric^15^ and breast cancer^16^.

A previous gene expression study has subdivided uveal melanoma in two distinct molecular classes with prognostic implications, as class 1 tumors are associated with a favorable prognosis^17^. A recent integrative study of uveal melanoma has further characterized four distinct classes, primarily based on chromosome 3 and chromosome 8 copy number alterations and secondarily based on gene alterations (*EIF1AX, SF3B1*), gene expression and methylation patterns^18^.

Unfortunately, many gene expression studies of uveal melanoma are small, limited by the rarity of the disease. Here, in addition to an analysis of 76 primary uveal melanoma samples using RNAseq, we present a meta-analysis of 196 patients with uveal melanoma which served as a training set for the discovery of gene expression patterns favoring liver metastasis in other tumor types.

## Results

We pooled three gene expression studies into a training dataset with 196 uveal melanoma patients and 13560 genes. For each gene, we fitted a univariable Cox survival model of relapse-free survival against expression separately in each dataset and computed a meta-analysis summary. This approach identified 656 genes negatively associated and 810 genes positively associated with relapse (FDR < 0.05). The Q-test for heterogeneity and visual inspection of forest plots did not show discrepancies between datasets. The top genes, ordered by FDR, are shown in Table 1 (detailed results in Table S1).

**Table 1:** Genes associated with relapse in uveal melanoma.

Gene	HR	95% CI	FDR	Location
*PTP4A3*	2.54	(2.01 to 3.20)	<0.001	8q24.3
*JPH1*	2.34	(1.87 to 2.94)	<0.001	8q21.11
*ID2*	0.48	(0.39 to 0.6)	<0.001	2p25.1
*ANG*	0.42	(0.33 to 0.55)	<0.001	14q11.2
*PRKDC*	1.97	(1.61 to 2.41)	<0.001	8q11.21
*POP1*	1.91	(1.57 to 2.33)	<0.001	8q22.2
*RAB11FIP1*	0.52	(0.42 to 0.63)	<0.001	8p11.23
*CDC25B*	2.06	(1.65 to 2.57)	<0.001	20p13
*CHAC1*	1.97	(1.59 to 2.43)	<0.001	15q15.1
*LPIN1*	0.51	(0.42 to 0.64)	<0.001	2p25.1
*CHD7*	1.97	(1.59 to 2.45)	<0.001	8q12.2
*VCPIP1*	1.99	(1.60 to 2.48)	<0.001	8q13.1
*ROPN1B*	0.50	(0.40 to 0.62)	<0.001	3q21.2
*MTFR1*	1.86	(1.52 to 2.28)	<0.001	8q13.1
*MCM4*	1.81	(1.49 to 2.19)	<0.001	8q11.21
*COL9A3*	1.88	(1.53 to 2.31)	<0.001	20q13.33
*ASAP1*	2.02	(1.61 to 2.55)	<0.001	8q24.22
*SPIRE1*	2.02	(1.60 to 2.55)	<0.001	18p11.21
*TLCD1*	1.83	(1.50 to 2.24)	<0.001	17q11.2
*RAB2A*	1.80	(1.48 to 2.18)	<0.001	8q12.1

Top 20 genes with lowest FDR shown (full results in the supplement) in a meta-analysis of univariable survival models against the expression of each individual gene.

A recent study of uveal melanoma^19^ identified 12 genes that have prognostic value. Eleven of these genes were available in the training dataset and for nine of them we confirmed the expected statistically significant positive (*EIF1B, ID2, LMCD1, MTUS1, ROBO1, SATB1)* respectively negative *(ECM1, HTR2B, RAB31)* association with relapse-free survival (all FDR < 0.05) in our meta-analysis. High expression of *BAP1*, suggestive of chromosome 3 disomy, was associated with a low risk of relapse (HR = 0.64, 95% 0.54–0.78, FDR < 0.001).

Many of the statistically significant genes in our meta-analysis resided either on chromosome 3 (N = 185) or on chromosome 8 (N = 183), together accounting for one fourth of all significant genes (25.1%, 369/1466). The log hazard ratio (HR) of genes from 3p and 3q was almost always negative, therefore higher expression, presumably in patients with chromosome 3 disomy, predicted longer relapse-free survival (mean log HR estimate −0.020, 95% −0.029 to −0.011, t-test P < 0.0001). The same was observed for genes from 6p (log HR −0.018, 95% −0.0305 to −0.006, P = 0.0033) and 6q (log HR −0.101, 95% −0.115 to −0.087, P < 0.0001). Finally, high expression of genes from 8q was associated with shorter relapse-free survival (P < 0.0001) while the inverse was observed for genes from 8p (P < 0.0001).

**Figure 1:**
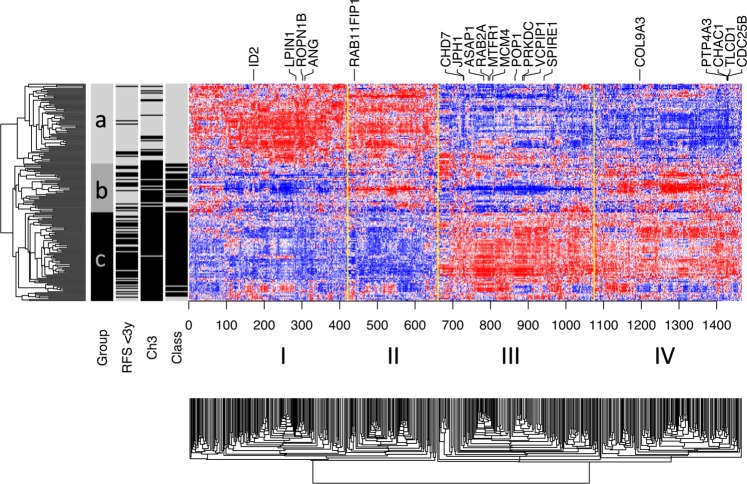
Gene expression heatmap. Hierarchical clustering with Pearson’s correlation similarity and average linkage. Heatmap showing the 1466 genes associated with prognosis in the meta-analysis of Cox models (FDR < 0.05). We perceive four main clusters (I-IV). In addition to favorable (**a**) and unfavorable (**c**) patient groups, there seems to be an intermediate group (**b**), characterized by a distinct pattern of low expression in cluster I and III and high expression in clusters II and IV. Chromosome 3 loss, class 2 tumors and relapse before 3 years of follow-up are shown in black.

The training data were pooled by computing standardized gene expression values (mean-centered and scaled to a variance of one). In Fig. 1, a heatmap of prognostic genes (FDR < 0.05) delineates four major gene clusters (I-IV). Gene expression within these clusters is roughly associated with molecular class, chromosome 3 status and relapse. The heatmap and tumor similarity tree also suggest the presence of three patient groups (a, b and c). All tumors from the top tier of the tree (subtree a) belong to class 1 (72 of 72 = 100%), shown in grey, and demonstrate rare relapses, shown in black. Inversely, the tumors from the high-risk group (subtree c) almost always belong to class 1 (74 of 80 = 92.5%)^17^, which is shown in black. In the middle, we see a group (subtree b) that is associated with intermediate risk of relapse (Fig. S1). This group is probably not adequately captured by the traditional two classes and appears to be biologically distinct. Tumors from the intermediate and high-risk groups mostly seem to differ on genes from cluster I and III, such as *CHD7, JPH1, ASAP1, POP1* and *PRKD*, which are highly inter-correlated (Pearson’s correlation P < 0.0001 for all pairs). An additional heatmap of all available genes can be found in the supplement (Fig. S2).

### Derivation of a liver metastasis score

Given the propensity of uveal melanoma for metastasis to the liver, we assumed that gene expression analysis could reveal features that predispose to liver metastasis in other cancer types. We further reasoned that it would be necessary to adjust for chromosome 3 loss, which is known to be associated with *BAP1* biallelic inactivation and metastatic risk in uveal melanoma. *BAP1* inactivation (mutation or loss) is a rare event in most cancer types and would be unlikely to be a universal predictor of liver metastasis. Therefore, we performed an additional meta-analysis. Within each dataset and for each gene we fitted multivariable Cox model of relapse-free survival that included gene expression and chromosome 3 status. In this approach, 63 genes were significantly correlated with relapse (FDR < 0.05). Again, the Q-test for heterogeneity and visual inspection of forest plots did not show discrepancies between datasets. The top 10 genes are shown in Table 2 (full results in Table S2). After adjustment for chromosome 3 status, the relation of genes residing on chromosome 3 with relapse was attenuated. Indeed, the expression of most chromosome 3 genes (777/782), including *BAP1* (HR = 0.779, 95% 0.620–0.977, FDR = 0.374), was not significantly associated with relapse-free survival when chromosome 3 status was included in the model. Twenty-eight significant genes (44% of all significant genes) were located on chromosome 8 and seven thereof in 8q24.3, the distal end of the chromosome 8.

**Table 2:** Genes associated with relapse in uveal melanoma.

Gene	HR	95% CI	FDR	Location
*JPH1*	2.00	(1.56 to 2.57)	<0.001	8q21.11
*PTP4A3*	2.13	(1.62 to 2.80)	<0.001	8q24.3
*ID2*	0.56	(0.44 to 0.70)	0.002	2p25.1
*POP1*	1.68	(1.37 to 2.06)	0.002	8q22.2
*CYP4X1*	1.62	(1.33 to 1.97)	0.004	1p33
*HPN*	1.65	(1.33 to 2.05)	0.009	19q13.11
*FYN*	0.61	(0.50 to 0.75)	0.009	6q21
*LPIN1*	0.58	(0.46 to 0.73)	0.009	2p25.1
*ETNPPL*	1.69	(1.34 to 2.12)	0.011	4q25
*MARC2*	0.63	(0.52 to 0.78)	0.012	1q41
*TLCD1*	1.68	(1.33 to 2.10)	0.012	17q11.2
*TSC22D1*	1.61	(1.30 to 1.99)	0.013	13q14.11
*VCPIP1*	1.69	(1.33 to 2.15)	0.014	8q13.1
*ANG*	0.52	(0.39 to 0.70)	0.014	14q11.2
*RAB11FIP1*	0.62	(0.50 to 0.77)	0.014	8p11.23
*DNAAF3*	0.56	(0.43 to 0.73)	0.014	19q13.42
*GALM*	0.67	(0.55 to 0.80)	0.014	2p22.1
*ASAP1*	1.70	(1.33 to 2.17)	0.015	8q24.22
*CBS*	1.48	(1.23 to 1.77)	0.016	21q22.3
*MTFR1*	1.61	(1.29 to 2.01)	0.018	8q13.1

Top 20 genes by lowest FDR shown (full results in the supplement) in a meta-analysis of bivariable survival models including chromosome 3 and the expression of each individual gene.

The pooled data of the training dataset were further used to build a prognostic scoring formula with the L1-penalized likelihood algorithm of the GLMnet package (supplemental methods) limited to the genes that were prognostic after adjustment for chromosome status 3 at FDR < 0.1 (Table S2). The procedure converged on a linear score based on the expression of only the two most significant genes, namely *JPH1* and *PTP4A3* (coefficients 0.147 and 0.249 respectively). In the training data, the two-gene prognostic score was significantly associated with progression-free survival in a multivariable survival model that included tumor class and chromosome 3 status (P < 0.0001). The prognostic score was also correlated with the expression of class genes (R^2^ = 0.66 against the mean of class 1 minus the mean of class 2 genes, P < 0.0001).

### Validation

#### Uveal melanoma

**Figure 2:**
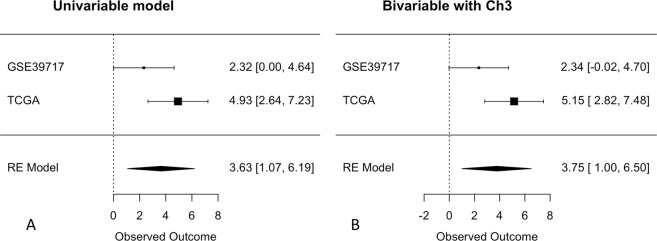
Forest plot of univariable (**A**) and bivariable (**B**) Cox regression of the continuous risk score in uveal melanoma. Log hazard ratio estimates with standard error bars are shown horizontally for both datasets (upper part) and the meta-analysis (RE model, bottom part). The lack of association is at zero (dotted vertical line). There was a positive association in both the univariable and bivariable model, both statistically significant in the meta-analysis.

The prognostic performance of the two-gene score was validated in independent public uveal melanoma data (GSE39717, N = 30 and the TCGA, N = 60). Meta-analysis of univariable Cox survival regression confirmed a statistically significant association with prognosis (Figs 2A and S3, log HR = 3.63, 95% 1.07–6.19, P = 0.0055). As expected, the score remained prognostic in a bivariable model including chromosome 3 status (Figs 2B and S3, log HR_score_ = 3.75, 95% 1.00–6.50, P = 0.0075).

#### Pan-cancer

**Figure 3:**
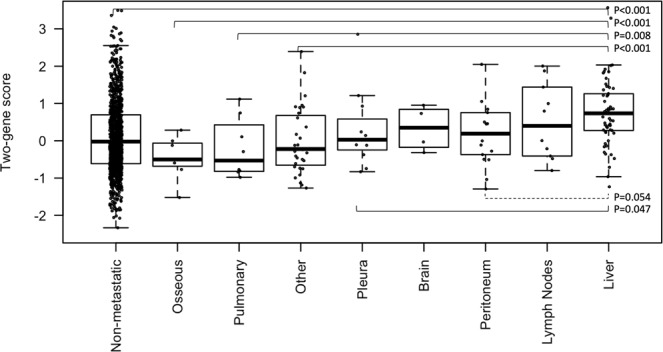
The two-gene score is highest in tumors with liver metastases. Data from the GSE2109 pan-cancer dataset. Statistically significant pairwise Wilcoxon’s comparisons are shown in solid lines (all values are FDR).

Our main objective was to examine whether the predisposition of uveal melanoma to liver relapse can be used to estimate the risk of liver metastasis in other malignancies, especially of epithelial origin. To this end, we examined the two-gene score values in a pan-cancer dataset (GSE2109) including 1181 primary tumors (1036 non-metastatic, 57 with liver metastases, 89 with other metastases). Most samples were obtained from colorectal (N = 274), breast (N = 248), endometrial (N = 147), ovarian (N = 132) and lung (N = 98) cancer. The risk score was significantly higher in tumors that developed liver metastases (t-test P < 0.0001, Fig. S4), a difference that persisted when we restricted the analysis to tumors that had metastasized (N = 146, t-test P < 0.0001, Fig. S4). The difference between tumors with and without liver metastasis also remained significant in a bivariable logistic model including the primary origin (logOR = 0.26, 95% 0.005–0.41, P = 0.043). A similar result is obtained when the score values are standardized per primary site (logOR = 0.29, 95% 0.03–0.55, P = 0.028). Looking at individual metastatic sites, the risk score was highest in the group with liver metastases and was statistically significant in pairwise comparisons with non-metastatic tumors but also against tumors harboring metastases in bone and lung (Fig. 3).

#### Breast cancer

**Figure 4:**
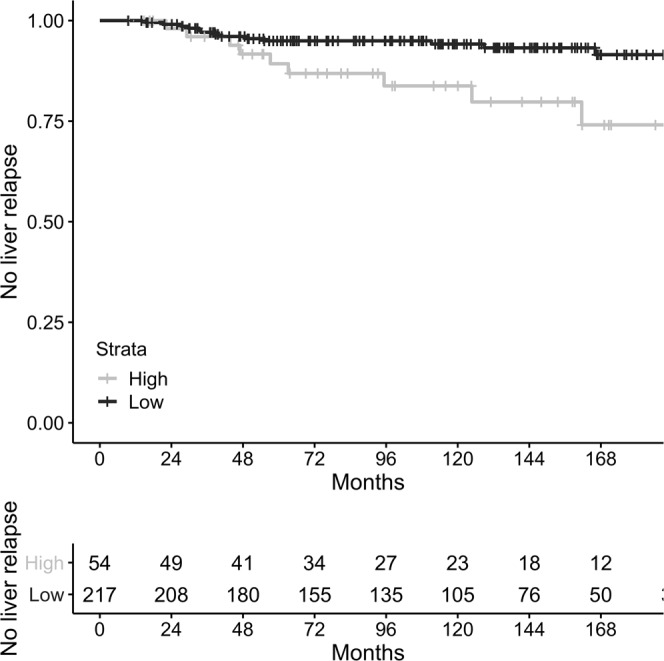
Kaplan-Meier curve of liver-first relapse in hormone-receptor positive breast cancer. Liver-first relapse occurred more often in the high-score group (P = 0.0098 for the continuous risk score). Data plotted using the top quintile as cutoff value (P = 0.011 at the specified cutoff).

We measured the expression of *PTP4A3* and *JPH1* in a breast cancer cohort by qPCR and used it to calculate the risk score in a series of 463 tumors for which the time and site of relapse were known (271 hormone receptor-positive, 86 triple-negative and 106 HER2-positive). The proportion of patients with liver-first relapses did not differ significantly (Fisher’s test P = 0.53) between HER2-positive tumors (10%), hormone receptor-positive (8%) and triple-negative tumor (6%). In hormone receptor-positive tumors, the two-gene prognostic score was associated with relapse (HR = 1.24, 1.04–1.47, P = 0.016) and specifically with first relapse to the liver (Fig. 4, HR = 1.50, 1.10–2.05, P = 0.0098) but not with relapse to non-liver sites (HR = 1.15, 0.94–1.41, P = 0.183). In triple-negative tumors, the two-gene score was not associated with relapse at any site (HR = 1.25, 0.77–22.4, P = 0.88) or liver-specific relapse (HR = 0.78, 0.29–2.08, P = 0.62). Similarly, in Her2 + tumors the score was not associated with relapse at any site (HR = 0.96, 0.70–1.32, P = 0.789, 38 events) or liver-specific relapse (HR = 0.99, 0.55–1.78, P = 0.981, 11 events).

#### Colorectal cancer

**Figure 5:**
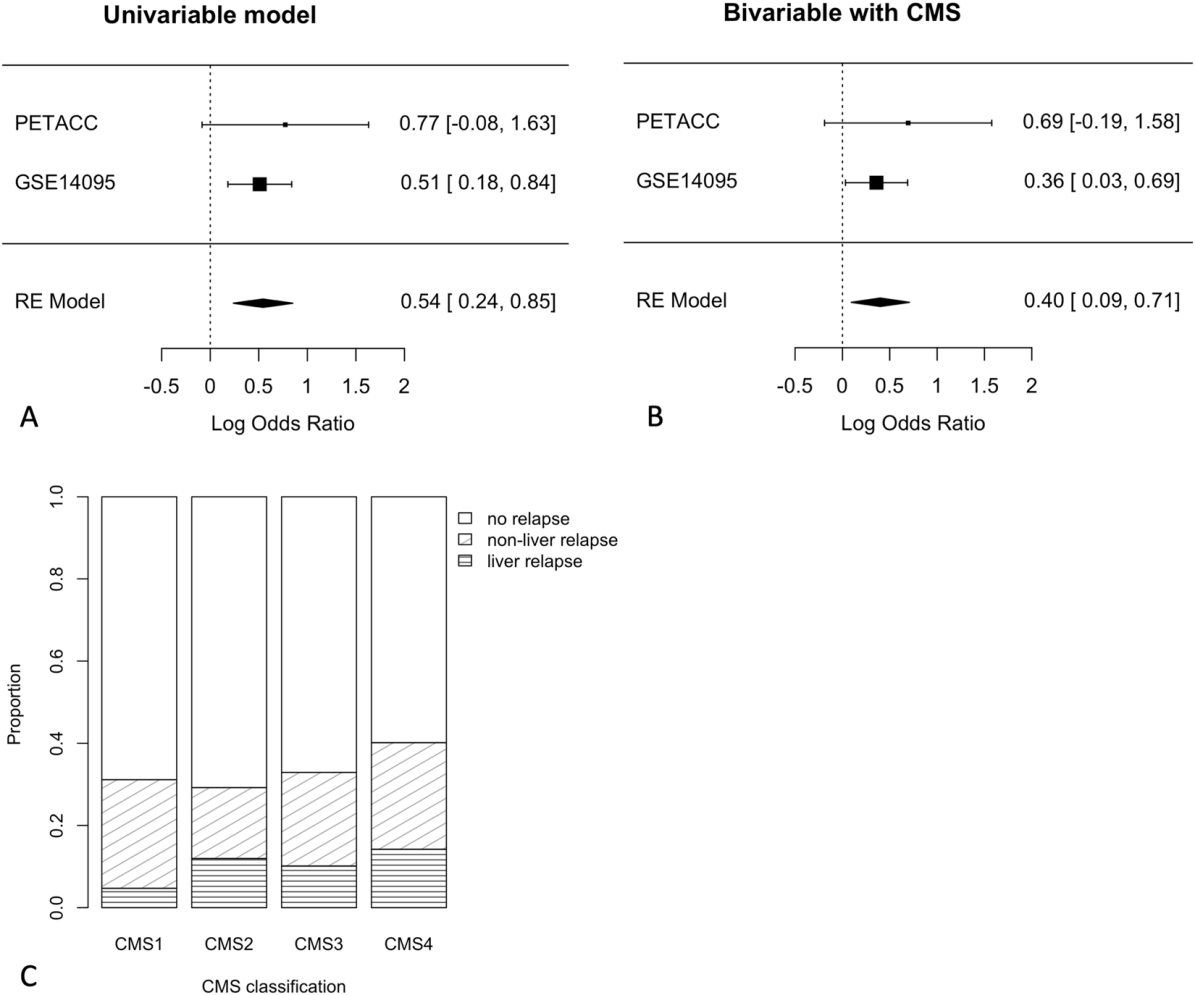
The risk score was specifically associated with liver relapse in colorectal cancer. Log hazard ratio estimates with standard error bars are shown horizontally for both datasets (upper part) and the meta-analysis (RE model, bottom part). The lack of association is at zero (dotted vertical line). In a meta-analysis (RE model) of **(A)** univariable and **(B)** bivariable logistic regression, the risk score was consistently and significantly associated with liver relapse, even after adjustment for CMS groups. Data from PETACC-3 and GSE14095. **(C)** In the prospective PETACC data, the proportion of total relapse was similar in CMS1-3, as expected. Liver relapse was lower in CMS1.

Finally, we used colorectal cancer data from the PETACC-3 trial (N = 604, 63 liver relapses) and a public retrospective series (GSE14095, N = 189, 53 liver relapses) to validate the risk score in colorectal cancer. In both datasets the risk score was significantly higher in patients with liver relapse (Wilcoxon’s test P = 0.047 and P = 0.011). In a meta-analysis of logistic regression models, a high-risk score was significantly associated with liver metastasis (Fig. 5A, logOR = 0.54, 95% 0.24–0.85, P = 0.0005). Interestingly, in the PETACC data, which also included non-liver relapses, the score was not associated with relapse in general (logOR = 0.00, 95% −0.57 to 0.57, P = 0.98), while relapse to non-liver sites was a negative trend (logOR = −0.65, 95% −1.39 to 0.06, P = 0.078).

The distribution of the risk score was visibly bimodal in both colorectal cancer datasets (Fig. S5), suggesting the presence of distinct sub-populations. Indeed, when tumors were separated by the consensus molecular subtype (CMS) classifier, the average risk score was highest in CMS2 and 4, and lowest in CMS1 and 3 (Fig. S5). The association between liver metastasis and the risk score remained significant in a bivariable model that included the CMS subtype (Fig. 5B, logOR = 0.40, 95% 0.09–0.71, P = 0.011). In the prospective PETACC data, the distribution of the risk score agreed with the proportion of liver relapses, which was lowest in CMS1 (Fig. 5C, logOR = −1.04 vs non-CMS1, 95% −2.11 to −0.19, P = 0.03). Liver relapse was only 13% of all relapse in CMS1, against 34%, 31% and 30% in CMS2–4 respectively. In the pooled colorectal cancer data, prediction of liver metastasis was improved by including both CMS and the risk score (ANOVA Chi^2^ test P = 0.0013 against a model including CMS only, P < 0.001 against the score only).

## Discussion

Different tumors have different patterns of metastatic spread^20,21^. Predicting the most likely site of relapse can help determine the most appropriate treatment and follow-up, such as scintigraphy for bone metastases and liver MRI for liver metastases. Early work has demonstrated that it is possible to distinguish tumors with metastatic potential from tumors that do not metastasize from their gene expression profile^22^. However, despite advances in our understanding of the cellular adaptations that occur in every distant site^23^, efforts to predict organ-specific metastasis are limited in size and scope. A small study of 20 colorectal tumors resulted in the identification of 37 genes that differed between tumors with and without liver metastases^24^. Another study of 123 colorectal tumors found 46 genes associated with liver metastasis^25^. Several of the genes, including *MMP1, MMP2, TIMP1* and *HIF1A*, are known cancer genes and facilitate stromal invasion. In a study of over 1000 breast tumors the most important risk factor for liver metastasis was the HER2-enriched subtype, but without more specific information at the gene level^26^.

A predictor of liver metastasis can be derived from any gene expression dataset with adequate follow-up. The advantage of using uveal melanoma is that its proclivity for liver metastasis is highly specific (~90%), meaning that it rarely metastasizes to other organs, and this proclivity cannot be explained by anatomical or vascular proximity. In that sense, tumors that behave biologically like uveal melanoma may be better candidates for liver-specific follow-up and interventions than tumors with a general tendency to colonize multiple organs, which would be at risk of extrahepatic recurrence.

We derived and validated a prognostic score that predicted relapse in uveal melanoma based on the weighted expression of only two genes (*PTP4A3* and *JPH1*) and applied it to the prediction of liver relapse in other malignancies. The observation that *PTP4A3* expression strongly correlates with metastatic risk in uveal melanoma is in agreement with previous studies of uveal melanoma^13^. In colorectal cancer, the *PTP4A3* gene regulates cancer cell adhesion and the expression of key ECM and adhesion genes^27^. *PTP4A3* also seems to facilitate metastasis through accumulation of MMP14^28^ and regulation of integrin β1^29^. Interestingly, copy number gains of *PTP4A3* at 8q are more frequent in colorectal tumors with liver metastases^30^ although this observation also includes broader alterations such as loss of 8p and gain of centromeric 8q^31^.

The *JPH1* gene codes junctophilin-1, a component of the junctional complex that binds the plasma membrane with the endoplasmic reticulum and regulates intracellular calcium^32^. Although *JPH1* has been found to be differentially expressed in metastatic versus non-metastatic uveal melanoma^33^ and thymoma^34^, its biological significance in cancer remains to be elucidated. Even in the absence of a direct causal relation, *JPH1* upregulation may reflect other upstream events, such as driver mutations in other genes or copy number gains in 8q21 involving nearby regulatory regions.

Our hypothesis was that the gene expression of uveal melanoma could identify biological predictors of liver-specific metastasis. In agreement with our initial hypothesis, the risk score developed in uveal melanoma was associated specifically with the risk of liver relapse. In the pan-cancer dataset (GSE2109) the score values were highest in tumors which developed liver metastasis, compared with tumors which metastasized to other sites. Although the number of events (liver metastases) is not sufficient to characterize individual tumor types, the relation between the two-gene score and liver metastasis remained significant in a multivariable model after adjustment for the site of the primary.

Metastasis to the liver is common in breast cancer^20^, but locoregional treatment such as surgical resection is only beneficial for carefully selected patients^35,36^, making their early identification essential. In hormone receptor positive breast cancer, the score was associated with metastatic liver relapse but not with relapse to other sites. The score was not predictive of liver relapse in triple negative or Her2+ breast cancer, a fact that could result from profound biological differences, significant heterogeneity in the case of triple negative tumors, or simply a lack of statistical power due to a small number of liver events in these cohorts. Her2+ breast tumors have a higher risk of liver metastasis^26^, but did not have higher score values in our study and may depend on the contribution of alternative biological pathways. For example, previous studies have shown that the expression of Claudin-2 is associated with early liver relapse in triple negative and hormone receptor positive breast cancer^37^.

The score was consistently associated with liver metastasis in a meta-analysis of two different colorectal cancer datasets. Other genes, such as *EREG, AREG* and *LCK* have also been associated with liver metastasis in a previous gene expression study of 160 colorectal cancer samples^38^ although it is unclear whether these genes are generally related with liver metastasis or are specific to colorectal cancer. CMS was also predictive of liver relapse in colorectal cancer, with CMS1 having a significantly lower proportion of liver relapses than CMS2–4. Given that the overwhelming majority of CMS1 tumors are right-sided, the influence of anatomical factors cannot be excluded, although a causal link between right-sidedness and low risk of liver metastasis is not obvious and cannot be inferred from our data. A lower incidence of liver metastases in right-sided tumors has also been observed previously^31^. Since both CMS and the risk score were independently predictive of liver metastasis in a bivariable model, integrating both in a clinically-oriented algorithm should further improve predictive performance.

Judging the reliability and, generally, the clinical performance (precision, accuracy etc) of the score at this stage is probably premature. First, the practical utility depends on the prior risk of liver metastasis, which varies considerably between cancer types^20^. It would be very hard to show a meaningful absolute increase in the probability of liver metastasis in cancer types which very rarely metastasize to the liver. Second, the overall risk of metastasis must also be estimated according to the known prognostic variables of each cancer type. For example, a T1N0 tumor would be very unlikely to spread to any organ. Finally, a specific cut-off would have to be chosen per tumor type but also depending on the clinical consequences. For example, even a modest increase in the estimated risk of metastasis could justify a liver MRI, while an invasive test or a therapeutic intervention would require a higher degree of confidence. A more detailed characterization of the score’s performance would be useful in a subsequent work that examines a precisely defined clinical context (disease type, risk factors, threshold for intervention etc).

Based on the above, our work provides proof-of-concept for predicting the risk of liver metastasis in hormone-sensitive breast cancer and colorectal cancer, based on a simple two-gene score. The performance could further be improved with the integration of type-specific information, such as the CMS classification of colorectal cancer. The results from a pan-cancer analysis indicate that the score could be useful in other tumor types and that it is highest in tumors with liver metastases, compared with tumors which colonize other sites. This suggests the existence of a common underlying biological process of liver metastasis that could be exploited for prognostic or therapeutic purposes. The elucidation of this mechanism presents an opportunity for further study.

## Methods

### Origin of samples

Untreated human samples from uveal melanoma and breast cancer were collected by the unit of surgical oncology from patients who provided written informed consent. The samples were stored in the Biological Resource Center (BRC) of the Institut Curie, France, in accordance with French regulations. The study was approved by the ethics committee and the institutional review board of the Curie Institute with approval number A10–024. All research was performed in accordance with relevant guidelines and regulations.

We searched for gene expression data from human primary samples of uveal melanoma in the Gene Expression Omnibus^39^ and manually curated datasets with adequate clinical annotation. At the time of retrieval, 18 data sets were screened and three were retained for analysis: GSE44295 (N = 57), GSE22138 (N = 63) and GSE39717 (N = 39, 30 with follow-up). The two largest datasets were used in conjunction with our own RNAseq data for model development (training dataset). We also downloaded publicly available RNAseq data from the TCGA (accessed on November 2017) for 80 patients, of which 60 had follow-up information and were retained for analysis. GSE39717 and TCGA data were used for validation (see Supplement Sections 1–3 and Fig. S6).

Gene expression data for colorectal cancer were obtained from the PETACC-3 study of adjuvant chemotherapy in colon cancer^40^, available in ArrayExpress (E-MTAB-990) and from a series of colorectal patients^41^ that were followed for the occurrence of liver metastases (GSE14095, N = 187). Finally, we also obtained a pan-cancer dataset which contained information on the site of relapse (GSE2109, N = 2158, 1181 of which with metastatic relapse).

We considered the use of the PanCancer TCGA data, based on the recent publication of curated clinical annotation^42^ (see Supplementary Table 1 in that article, columns T and U). Unfortunately, despite the inclusion of over 10,000 patients, the occurrence of liver events was recorded for only 262 patients, raising concerns about the quality of the follow-up with respect to that specific outcome. For example, none were documented for patients with colorectal cancer (N = 618), a fact that limited the usefulness of this resource for our purposes.

Inactivation of *BAP1* is common in uveal melanoma but can also occur in kidney cancer^7^, mesothelioma^8^ and cholangiocarcinoma^9^. However, mesothelioma and cholangiocarcinoma are rare and we could not find gene expression datasets documenting liver metastasis, not just overall survival or relapse-free survival, which could be used our purposes. In addition, cholangiocarcinoma is in immediate proximity to the liver and would not be a useful model of liver metastasis, more likely reflective of local invasion instead. Out of 33 patients with renal cancer in the pan-cancer GSE2109 dataset only 10 had distant metastases and none had liver metastases, making any kind of statistical analysis impossible.

### Total RNA extraction

Total RNA was extracted from fresh frozen uveal melanoma and breast cancer samples with the acid-phenol guanidium method. The quantity of RNA was assessed by using an ND-1000 NanoDrop Spectrophotometer with corresponding software (Thermo Fisher Scientific Inc., Wilmington, DE). RNA quality was determined by agarose gel electrophoresis.

### RNAseq

Library preparation was done using the Illumina TruSeq kit according to manufacturer’s instructions. Libraries were sequenced to produce paired-end reads. We used STAR v2.5^43^ to align against the GRCh37 DNA primary assembly and the Ensembl annotation r75 (available from ftp://ftp.ensembl.org/pub/release-75/). Quality control was done with RNA-SeQC (v1.1.8)^44^ and custom scripts.

The raw counts were estimated with the Rsubread package (v1.2) and then imported by edgeR (v3.12)^45^. Raw counts were filtered by only keeping genes with at least 1 count-per-million in at least 5 samples, normalized with the trimmed mean of M-values (TMM) and log-transformed, according to the edgeR recommendations.

### Real-time PCR

The RT-qPCR method, including cDNA synthesis and PCR conditions, has been previously described in detail^46^. We quantified transcripts of an endogenous RNA control gene involved in cellular metabolic pathway, namely TBP (Genbank NM_003194), which encodes the TATA box-binding protein (a component of the DNA-binding protein complex TFIID)^47^. Each sample was normalized on the basis of its TBP content and against a median of 10 normal breast tissue samples, with the ΔCt method.

### Data analysis and statistics

Statistical analyses were done in R version 3.5. All statistical tests were two-sided and considered significant if P < 0.05. P-value adjustment for multiple comparisons was used to control the false discovery rate^48^. Survival models were built with the *survival* R package^49^ and the *coxph()* function. By default, this function fits a Cox proportional hazards regression model with the Efron approximation for tied event times.

We used the metafor package with the random effects model and the default restricted maximum-likelihood estimator of residual heterogeneity (τ^2^) to perform meta-analyses^50^. Specifically, for each dataset and each gene the log-transformed hazard ratio estimate (yi) and its associated standard error (sei) were extracted from a univariable and bivariable (with chromosome 3 status) model. The rma() function from the metafor package was then called with *rma(yi, sei)*. The Q-test was used to test for significant heterogeneity.

The nearest-neighbor chain algorithm was used for hierarchical clustering (reviewed in^51^). Pearson’s correlation was used as a similarity metric with average linkage. We used the *nclust* package (v2.0.1) for this purpose, available from https://bcf.isb-sib.ch/nclust/.

The coxnet method^52^ in the GLMnet package was used to derive a linear prognostic score. The development of the prognostic score was in accordance with the REMARK recommendations.

### Gene expression estimate of chromosome 3 status

Over all uveal melanoma datasets, the chromosome 3 status had been estimated by dedicated methods (aCGH or FISH) for 119 patients. For the remaining patients, we inferred the chromosome 3 status from gene expression data. Using patients with known chromosome 3 status, we trained a binomial (loss/no loss) GLMnet model on 95 patients and validated it on the remaining 24 patients. Data from the 24 patients were not considered during the derivation of the model. Only the expression of genes residing on chromosome 3 was used, as these genes would be directly affected by a loss of chromosome 3.

Validation on the remaining 24 patients demonstrated a very high concordance (23/24 = 96%) of gene expression estimates with other methods (FISH, aCGH). The same model was subsequently applied to 77 patients for whom the chromosome 3 status was missing. The gene expression prediction of chromosome 3 status was significantly associated with relapse-free survival (HRmonosomy 4.30, 95% 2.34–7.88, P < 0.0001). The proportion of samples with monosomy did not differ significantly by estimation method (FISH, aCGH or gene expression, Fisher’s test P = 0.519).

## Supplementary information

Supplement with changes marked

Supplementary Table 1

Supplementary Table 2

## Data Availability

The RNAseq data produced by the study can be found in the European Genome-phenome Archive (accession EGAS00001002932). Public data used in this work: https://www.ncbi.nlm.nih.gov/geo/query/acc.cgi?acc=GSE44295 https://www.ncbi.nlm.nih.gov/geo/query/acc.cgi?acc=GSE22138 https://www.ncbi.nlm.nih.gov/geo/query/acc.cgi?acc=GSE39717 https://www.ncbi.nlm.nih.gov/geo/query/acc.cgi?acc=GSE2109 https://www.ebi.ac.uk/arrayexpress/experiments/E-MTAB-990/ https://portal.gdc.cancer.gov/projects/TCGA-UVM
