# Supplementary material for: Prospective validation in epithelial tumors of a gene expression predictor of liver metastasis derived from uveal melanoma
Source: Sci Rep. 2019 Nov 20;9:17178. doi: 10.1038/s41598-019-52841-y (PMC6868129; doi:10.1038/s41598-019-52841-y)

Prospective validation in epithelial tumors of a gene expression predictor of liver metastasis derived from uveal melanoma.

Petros Tsantoulis,[1,2,3*] Mauro Delorenzi,[3,4,5] Ivan Bièche,[6] Sophie Vacher,[6] Pascale Mariani,[7] Nathalie Cassoux,[7] Alexandre Houy,[6,8] Marc-Henri Stern,[6,8] Sergio Roman-Roman,[9] Pierre-Yves Dietrich,[1,2] Arnaud Roth,[1,2] Wulfran Cacheux[10,1,6]

1. Hôpitaux Universitaires de Genève, Service d'Oncologie, Geneva, Switzerland
2. University of Geneva, Geneva, Switzerland
3. SIB Swiss Institute of Bioinformatics, Bioinformatics Core Facility, Lausanne, Switzerland
4. University Lausanne, Department of Fundamental Oncology, Lausanne, Switzerland
5. Ludwig Institute for Cancer Research, Epalinges, Switzerland
6. Institut Curie, Département de génétique, Paris, France
7. Institut Curie, Département de chirurgie, Paris, France
8. Institut Curie, PSL Research University, INSERM U830, Paris, France
9. Institut Curie, PSL Research University, Translational Research Department, Paris, France
10. Hôpital Privé – Pays de Savoie, Oncology department, Annemasse, France

Supplementary methods

1. Processing of array expression data

Both processed and raw data, when available, were imported from the GEO and ArrayExpress. Raw Affymetrix data were treated with the RMA algorithm from the “affy” BioConductor (v3.4) package using the default settings (1).

Scaling of different datasets was performed with the scale() function from base R to a mean of zero and a variance of one. This was done in order to make distributions from different technologies (microarray, RNAseq) comparable, so that a single score equation can be used across datasets without modification. Otherwise the coefficients of the gene score would have to be re-calculated (and validated) separately for each dataset.

2. Molecular classes

Based on the molecular classification of uveal melanoma into two classes by Onken *et al.* (2), we used their published list of 60 class-specific genes to assign the most likely class to each tumor. The distribution of the difference between the means of the expression of class 1 and the mean of class 2 genes, was clearly bimodal (supplementary figure 7) and was used to classify the tumors into the two classes. Visual inspection of the gene expression heatmap (supplementary figure 8) further supported this classification.

3. Univariable and bivariable survival models

For the purposes of the meta-analysis in uveal melanoma, the Cox survival models were specified as follows:

Univariable: coxph(Surv(interval, event) ~ exp[x,])

Bivariable: coxph(Surv(interval, event) ~ chr3 + exp[x,])

where chr3 is a factor (categorical variable) with two levels (loss/no loss) and exp[x,] is the standardized expression of gene x, within each dataset.

4. Consensus Molecular Subtypes (CMS) of colorectal cancer

The molecular classification of colorectal cancer has been published previously(3) and an open-source reference classifier is available.[1] This classifier was used to classify PETACC and GSE14095 data based on both the RandomForest and SSP methods. Whenever the RandomForest failed to assign a CMS class, the SSP algorithm was used. The proportion of CMS groups was similar to previously published results.

5. Derivation of a prognostic linear model

The pooled data of the model-fitting dataset (training, N=196 patients) were used to train a prognostic model with the penalized likelihood algorithm of the GLMnet package (coxnet method). The following parameters were used for the derivation of a linear predictor: nfolds=16, to increase the number of cross-validation folds, and maxit=200000, to increase the iterations that are allowed until convergence can be achieved. The random generator

[1] https://github.com/Sage-Bionetworks/CMSclassifier

seed was fixed in order to ensure reproducibility. To improve the chances of convergence on a parsimonious model, the algorithm was applied only on candidate genes, which were associated with survival after adjustment for chromosome 3 status at FDR<0.1 in the meta-analysis (N=119, S. Table 2).

The linear predictor was trained to correlate with the risk of relapse. The predictor is a linear combination of two variables p and j, corresponding to the standardized expression of *PTP4A3* and *JPH* respectively. The score S is calculated with the following formula:

$$S = 0.249 * p + 0.147 * j$$

The resulting values are mean-centered and standardized within each dataset.

In order to verify the robustness of the model-generating procedure, we also performed 100 consecutive runs without fixing the random number generator seed. Genes *PTP4A3* and *JPH1* were included in all the resulting predictive models.

References

1. Irizarry RA, Bolstad BM, Collin F, Cope LM, Hobbs B, Speed TP. Summaries of Affymetrix GeneChip probe level data. Nucleic Acids Res. 2003 Feb;31(4):e15.
2. Onken MD, Worley LA, Ehlers JP, Harbour JW. Gene expression profiling in uveal melanoma reveals two molecular classes and predicts metastatic death. Cancer Res. 2004 Oct;64(20):7205–9.
3. Guinney J, Dienstmann R, Wang X, de Reyniès A, Schlicker A, Soneson C, et al. The consensus molecular subtypes of colorectal cancer. Nat Med. 2015 Oct;21(11):1350–6.

Figure S1

Kaplan-Meier plot of subgroups from figure 1B. The differences between a-vs-b and a-vs-c are significant (Cox regression, P-values shown below).

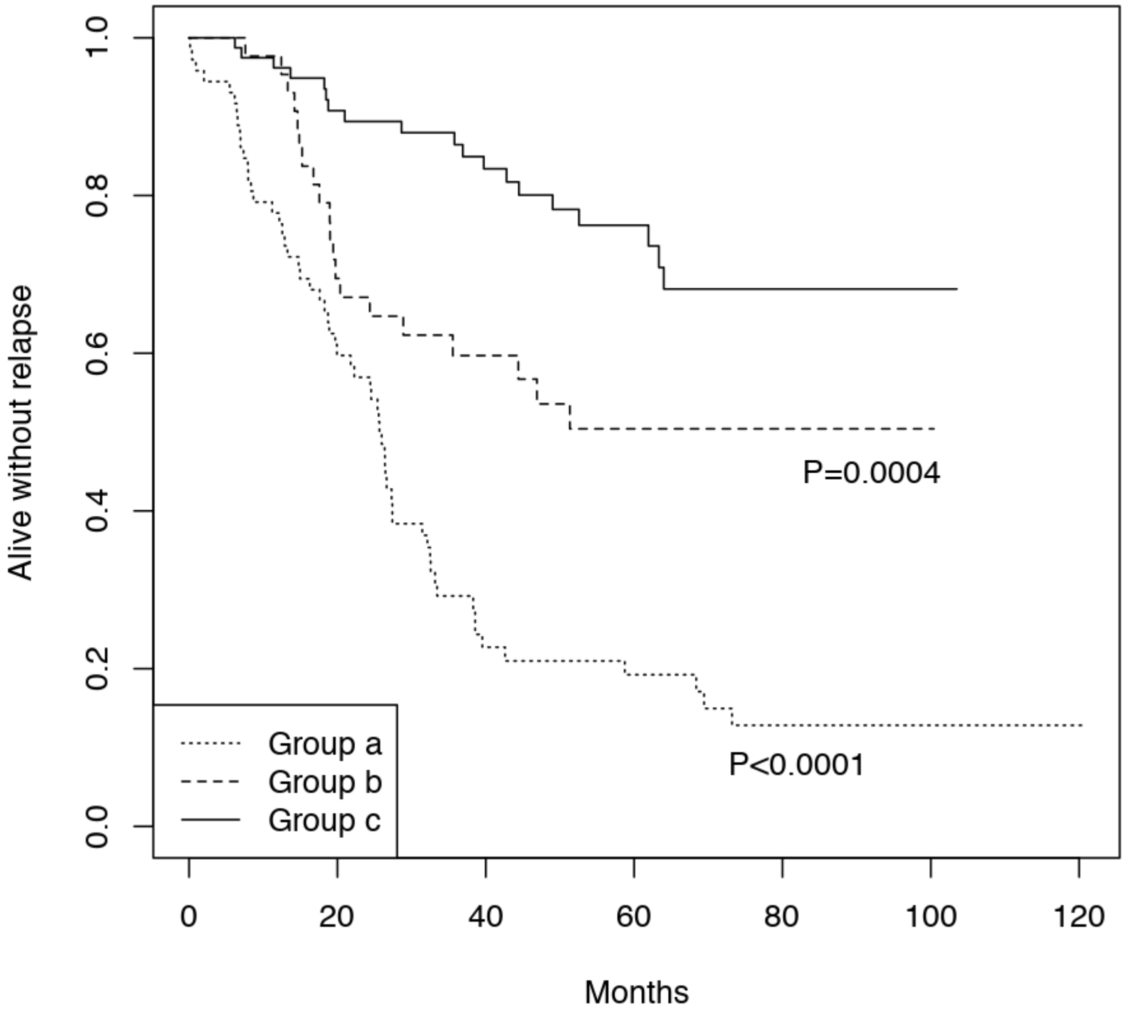

Figure S2

Hierarchical clustering with Pearson's correlation similarity and average linkage. Heatmap of all available genes. The vertical color bars to the left show patients with RFS of less than 3 years, chromosome 3 monosomy and molecular class (all in black). The most significantly prognostic genes in the meta-analysis are shown for chromosome 8 (twenty genes in light green) and other chromosomes (twenty genes in blue). The horizontal color bar below the heatmap is grey for genes that are not significantly prognostic and black for FDR<0.05.

It can be seen that genes *PTP4A3* and *JPH1* belong to distinct clusters, probably corresponding to associated pathways. *BAP1* (in red) is also distinct and centered on a narrow gene cluster.

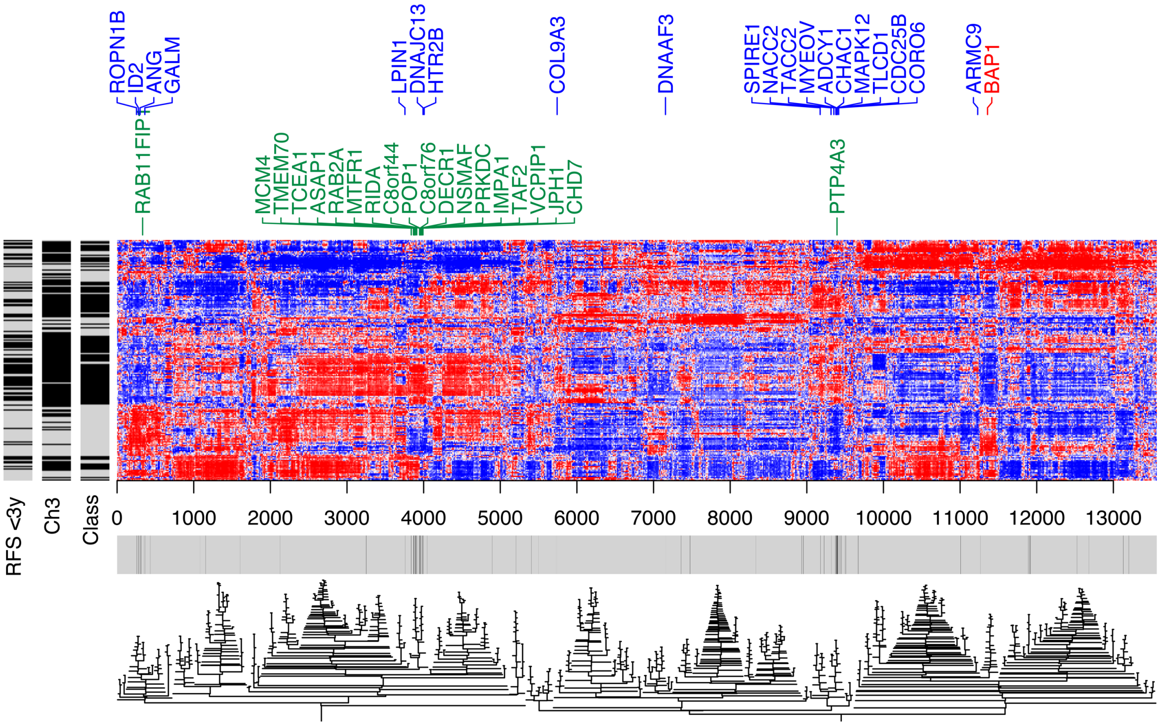

Figure S3

Risk score and relapse-free survival in uveal melanoma (validation data). Pooled data from GSE39717 and TCGA. The score was separated at a median cutoff for plotting.

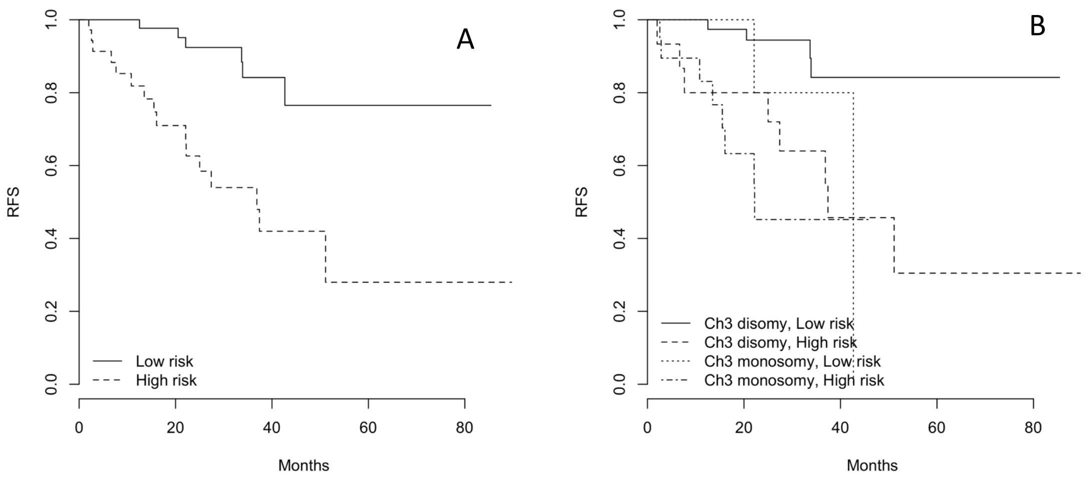

Figure S4

Boxplot of the risk score in tumors from the pan-cancer dataset. A. Comparison of tumors without liver metastasis (including non-metastatic tumors and tumors with other metastases) with tumors that presented liver metastases. B. Comparison of tumors with liver metastases with metastatic tumors that presented metastases in other sites.

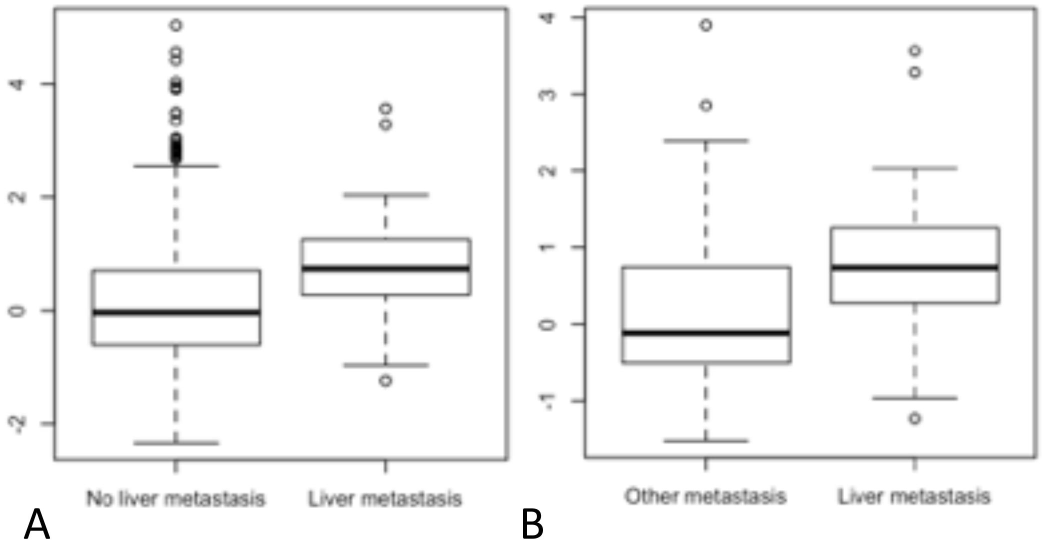

Figure S5

Distribution of the risk score in colorectal cancer. A bimodal distribution of the risk score is observed in both datasets (PETACC and GSE14095). Significant differences can be seen between the four CMS groups in the lower part of the figure (Wilcoxon's test, with Holm-Bonferroni P-value adjustment for multiple hypothesis testing).

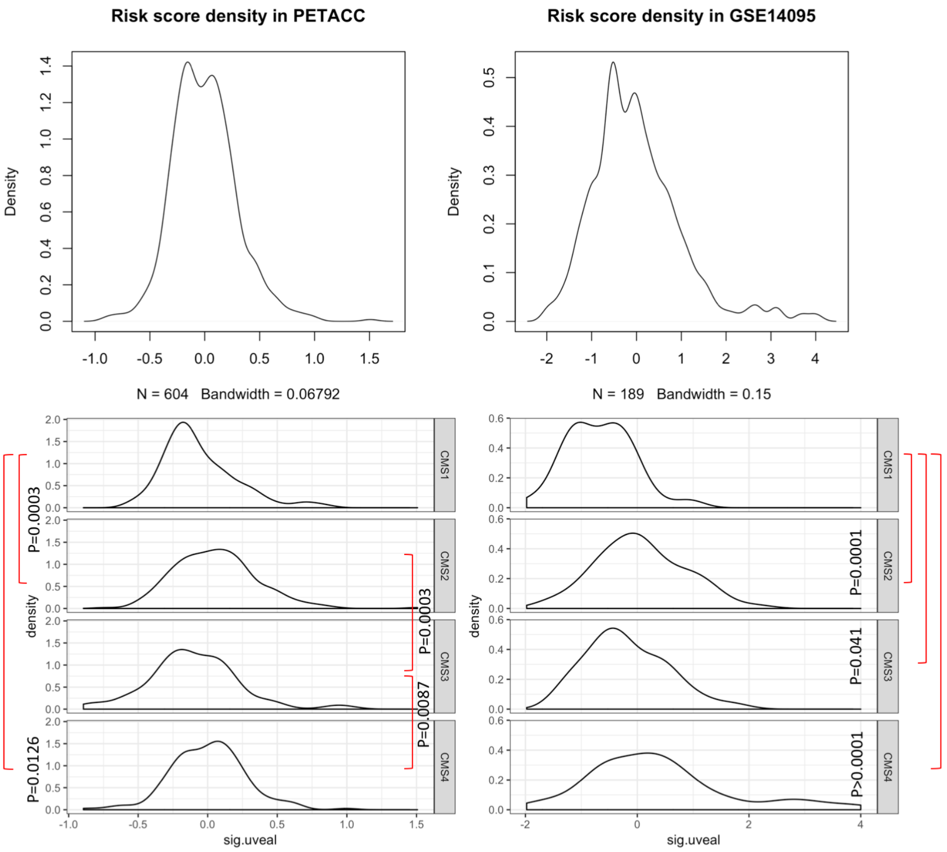

Figure S6

Data flow chart. A summary of the different datasets that have been used and their role in the training and validation process.

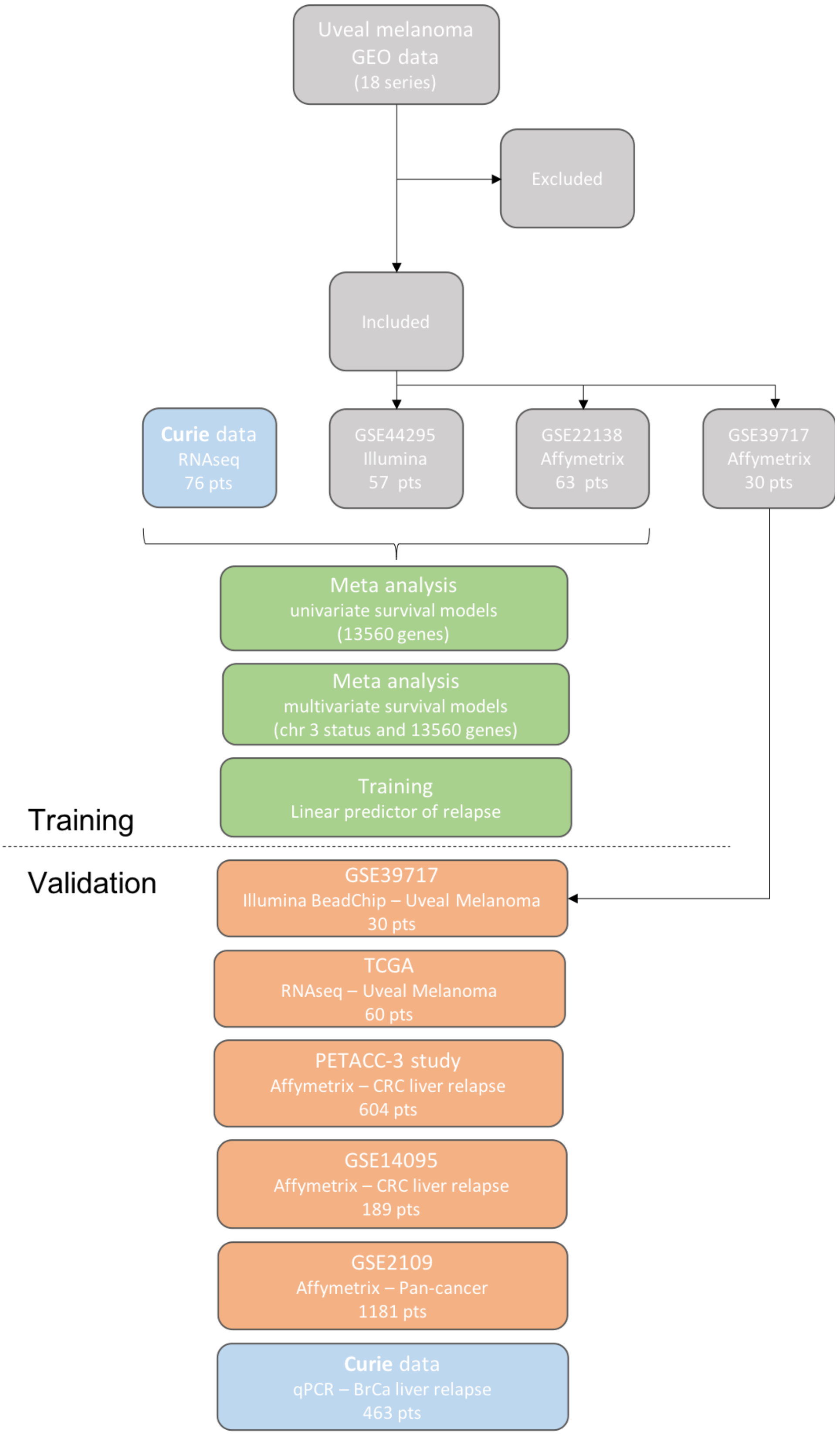

Figure S7

Distribution of the molecular class gene expression. The values visibly separate the tumors into two distinct peaks, corresponding to the two molecular classes.

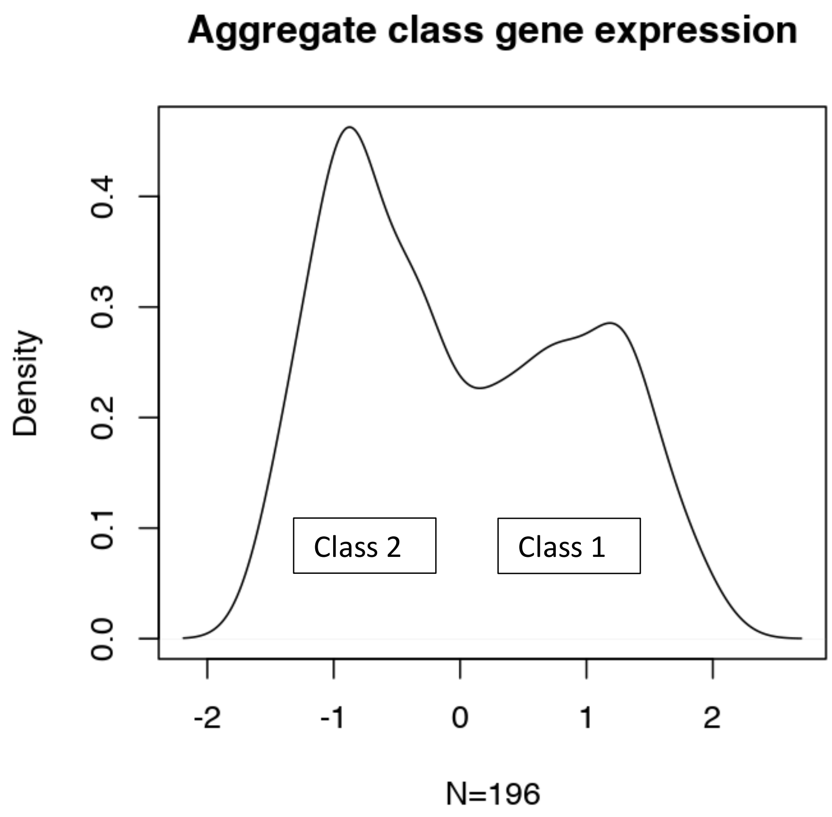

Figure S8

Heatmap of genes that have been associated with Class 1 and Class 2 tumors. The color bar to the left corresponds to predicted molecular class (red=class 1, deep blue=class 2).

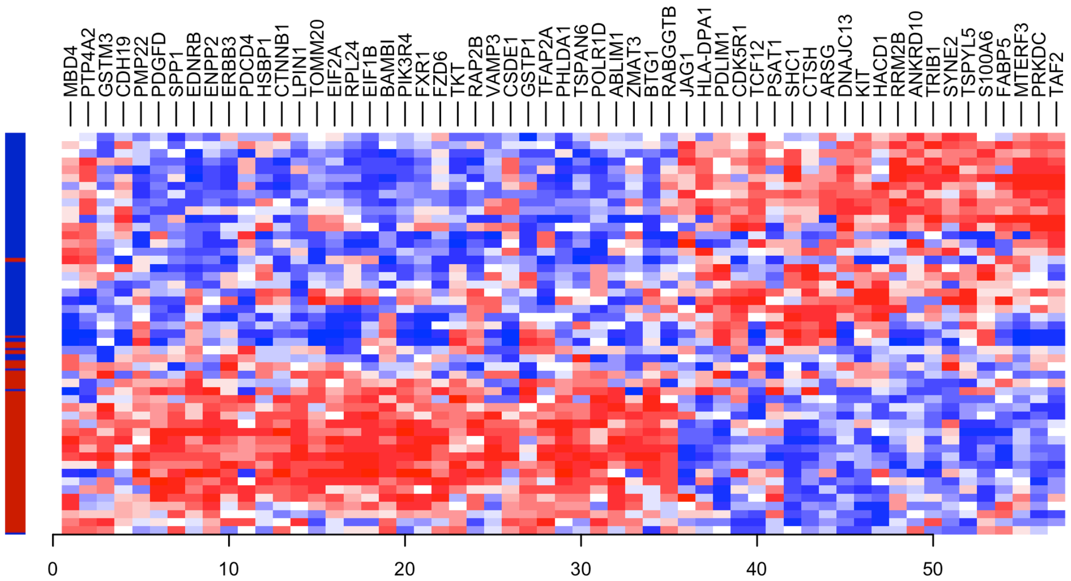

Supplement: Supplementary file 1 — Supplement with changes marked [file 41598_2019_52841_MOESM1_ESM.pdf]
